# Only Minor Complications Are Reported After Needle Arthroscopy: A Systematic Review

**DOI:** 10.1016/j.asmr.2025.101158

**Published:** 2025-04-28

**Authors:** Alex B. Walinga, Thijs van der Stappen, Gino M.M.J. Kerkhoffs, Kaj S. Emanuel

**Affiliations:** Department of Orthopedic Surgery and Sports Medicine, Amsterdam University Medical Center, University of Amsterdam, Amsterdam, The Netherlands; Amsterdam Movement Sciences, Sport, Musculoskeletal Health, Amsterdam, The Netherlands

## Abstract

**Purpose:**

To assess the incidence, etiology, and severity of complications after needle arthroscopy of joints.

**Methods:**

The review protocol was preregistered with PROSPERO (CRD42023443809) and conducted in line with the Preferred Reporting Items for Systematic Reviews and Meta-analyses guidelines. Multiple libraries were systematically searched to identify articles that reported on the complication rate after needle arthroscopy. The quality of evidence was assessed using the Risk of Bias in Non-randomized Studies of Interventions tool. The primary outcome measure was the incidence rate of complications after needle arthroscopy, with secondary outcomes including nature and severity of complications.

**Results:**

Eleven articles were included in the final analysis, totaling 1,624 patients. The overall reported complication rate after needle arthroscopy ranged between 0% and 9.68% depending on the joint: ankle, 0% to 9.68%; knee, 0% to 8.33%; and shoulder, 0% to 3.3%. Vasovagal reactions were the most frequently reported complication, presumably caused by needle phobia. All complications were classified as grade I according to the Clavien-Dindo-Sink classification.

**Conclusions:**

In this study, we found that the reported complication rate after needle arthroscopy ranged from 0% to 9.68%. All reported complications were classified as grade I according to the Clavien-Dindo-Sink classification.

**Level of Evidence:**

Level IV, systematic review of Level III and IV studies.

Needle arthroscopy has gained increased popularity in the treatment of joint pathologies. This minimally invasive arthroscopic technique facilitates both diagnostic and therapeutic interventions, with the possibility of being performed under local anesthesia outside the traditional operating theater.^1^, ^2^, ^3^, ^4^, ^5^ In the late 1980s, needle arthroscopy was introduced to minimize invasiveness of diagnostic arthroscopies and (small surgical) interventions.^6^ Comparing that with recent years, the image quality of needle arthroscopes has improved and the tool set has been expanded.^7^ This has led to a swift increase in indications that reportedly align with the capabilities of needle arthroscopy.^5^^,^^8^^,^^9^ In addition, studies have shown the equivalence of needle arthroscopy to conventional arthroscopy with improved functional outcomes and better cost-effectiveness in the treatment of several intra-articular pathologies, such as partial meniscectomy or detection of tears in articular cartilage.^10^, ^11^, ^12^, ^13^, ^14^ All these developments have resulted in an increase in popularity of needle arthroscopy.

However, the procedure’s safety is a concern as always with emerging techniques. We know from conventional arthroscopy that the use of this technique is not without risks, although the complications are infrequent (1.5%-11%) and mostly minor.^15^^,^^16^ The most frequently reported complications were neurologic injuries.^15^^,^^16^ Despite the relatively small equipment and less invasive nature of needle arthroscopy, the potential impact of an outpatient setting and differences in handling and visual feedback may influence the rate or nature of complications and, therefore, needle arthroscopy may not be comparable to conventional arthroscopy. Consequently, the purpose of this systematic review was to assess the incidence, etiology, and severity of complications after needle arthroscopy of joints. We hypothesized that needle arthroscopy would show a low complication rate overall but that its unique characteristics—such as outpatient use and local anesthesia—may give rise to a distinct complication profile.

## Methods

The protocol for this systematic review was preregistered with the International Prospective Register of Systematic Reviews (PROSPERO) (CRD42023443809) before data collection.^17^ The Preferred Reporting Items for Systematic Reviews and Meta-analyses statement was followed as a guideline for this study.^18^ In consultation with a medical librarian, a search strategy was formulated and conducted. We searched eligible studies using PubMed, Embase, and the Cochrane Library for studies published before November 2023 using the following keywords: needle arthroscop∗, in-office arthroscop∗, office based arthroscop∗, and nanoarthroscop∗, including synonyms and Medical Subject Headings (MeSH) terms (Appendix Table 1 shows the full search syntax per library). All given search results were independently screened by 2 researchers (A.B.W., T.v.d.S.) on the title and abstract. Subsequently, full texts were screened. Discrepancies in study selection were resolved by a consensus meeting with a third researcher (K.S.E.). In addition, forward and backward citations were screened for eligible studies.

Inclusion criteria encompassed studies involving a minimum of 5 patients, with a mean age of at least 16 years, who underwent treatment with a needle arthroscopic intervention in one of the appendicular joints. Needle arthroscopy was defined as a minimally invasive procedure used to diagnose and treat joint issues with small incisions using a needle-like device with a camera and maximum diameter of 2.5 mm. The studies had to report whether complications occurred during or after the interventions. Studies in which needle arthroscopy was directly followed by conventional arthroscopy were excluded because it could be unclear whether complications arose due to either arthroscopy. Data from joints analyzed in fewer than 3 studies were excluded from this review. Additionally, if data for a specific joint were reported separately within a study, only those data were excluded, not the entire study. Studies were excluded if they were not written in English or had the following study designs: systematic reviews, case reports, cross-sectional studies, technique articles, cadaveric studies, animal studies, editorials, and test-tube laboratory research studies.

Two independent reviewers (A.B.W., T.v.d.S.) assessed the methodologic quality of the included studies using the Risk of Bias in Non-randomized Studies of Interventions (ROBINS-I) tool.^19^ The ROBINS-I tool is designed to assess the risk of bias in studies that did not use randomization to allocate interventions.

The primary outcome of this review was the incidence rate of complications that occurred during or after an intervention with a needle arthroscope. The term “complication” was defined according to the definition of Sokol and Wilson^20^: “any undesirable, unintended, and direct result of an operation affecting the patient.” The secondary outcome variables were the nature and severity of these complications. The severity of the complications was assessed with the help of the modified Clavien-Dindo-Sink complication classification system for orthopaedic surgery.^21^ Additionally, we extracted the following per study: author, title, year of publication, country, study type, treatment used, type of needle arthroscopy, anesthesia used (i.e., general, regional, or local), inclusion period, and follow-up duration. Patient characteristics were also extracted: sex, age, diagnosis, and joint.

The complication rate was defined as the proportion of complications to the total number of interventions. Data were presented for each of the different joints and plotted without pooling using a forest plot in R (version 4.4.1) via the meta-package.^22^ Data were extracted independently by 2 reviewers (A.B.W., T.v.d.S.) using Microsoft Excel (version 16.52; Redmond, WA).

## Results

**Fig 1:**
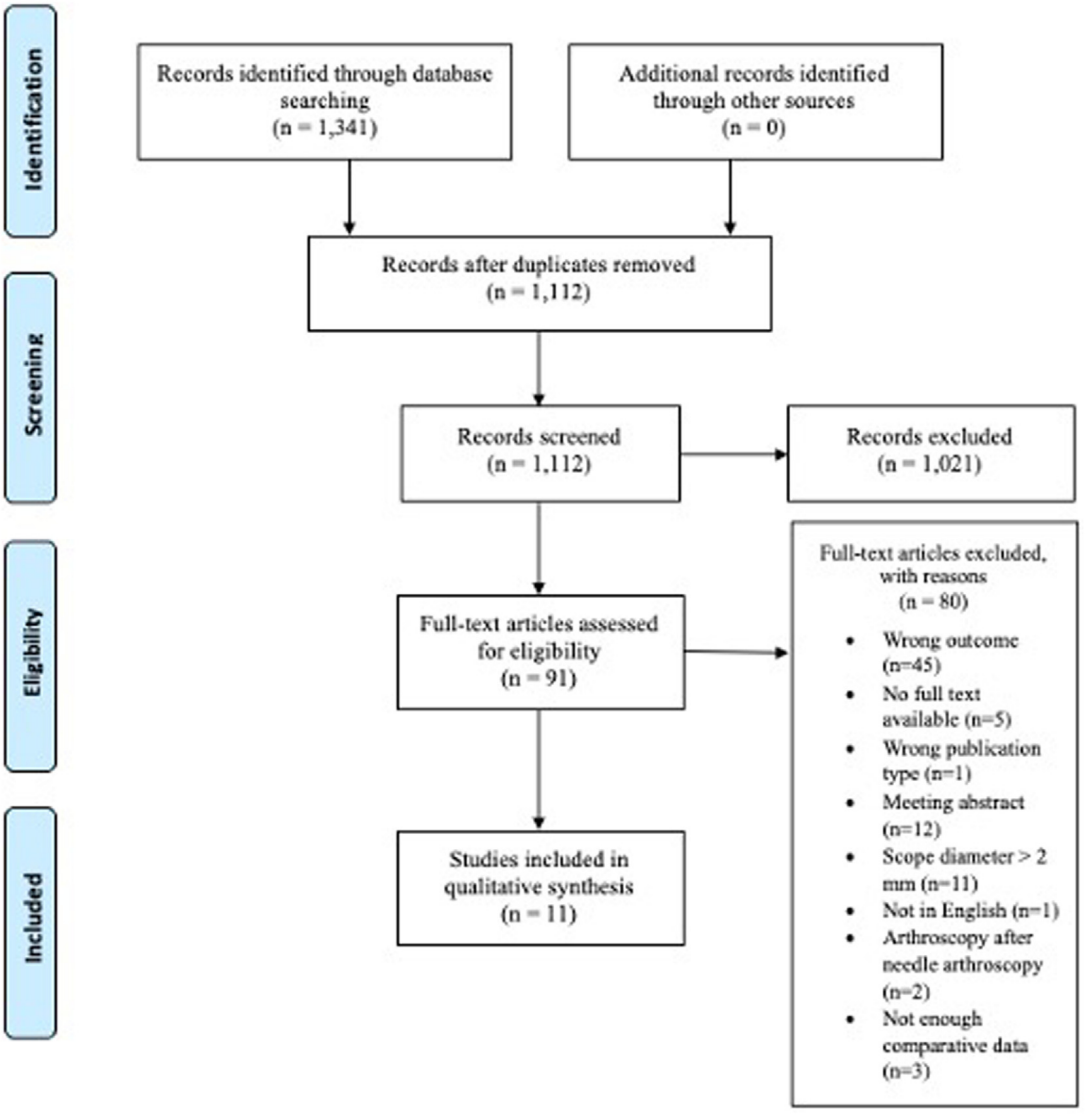
Preferred Reporting Items for Systematic Reviews and Meta-analyses flowchart. (n, number of articles.)

After duplicates were deleted, the initial search strategy yielded 1,112 studies, of which 11 articles were deemed eligible for inclusion (Fig 1). Additional forward and backward citation screening did not yield further studies for inclusion. Most of the studies (n = 9, 82%) were published after 2020. Of the 11 studies, 9 (82%) used a retrospective study design whereas 2 (18%) were case series. No randomized controlled trials meeting the inclusion and exclusion criteria were identified (Table 1). The risk of bias was mostly influenced by bias due to confounding and selection bias. A full evaluation of the ROBINS-I tool can be found in Appendix Table 2.

**Table 1:** Quality Assessment of Included Studies Using ROBINS-I Tool

Authors	Year	Journal	Level of Evidence	Study Design	ROBINS-I Assessment
Andreozzi et al.^25^	2022	*Arthroscopy, Sports Medicine, and Rehabilitation*	IV	Retrospective cohort study	Low
Annibaldi et al.^26^	2022	*Journal of Experimental Orthopaedics*	IV	Retrospective cohort study	Moderate
Colasanti et al.^27^	2022	*Arthroscopy: The Journal of Arthroscopic and Related Surgery*	IV	Retrospective cohort study	Moderate
DeClouette et al.^28^	2022	*Cureus*	IV	Retrospective cohort study	Moderate
Lopas and Mir^29^	2023	*The Iowa Orthopedic Journal*	IV	Retrospective cohort study	High
McMillan et al.^23^	2019	*Orthopaedic Journal of Sports Medicine*	IV	Retrospective cohort study	Low
Mercer et al.^30^	2022	*Arthroscopy, Sports Medicine, and Rehabilitation*	IV	Retrospective cohort study	Moderate
Moreland et al.^31^	1995	*Journal of Clinic Rheumatology*	IV	Retrospective cohort study	Moderate
Schaver et al.^13^	2023	*Journal of Orthopaedics*	III	Retrospective cohort study	High
Stornebrink et al.^24^	2021	*Journal of Experimental Orthopaedics*	IV	Case series	Low
Stornebrink et al.^32^	2022	*World Journal of Orthopedics*	IV	Case series	Low

ROBINS-I, Risk of Bias in Non-randomized Studies of Interventions.

**Table 2:** Study Characteristics of All Included Studies

Authors	Year	County	Inclusion Period, mo	No. of Patients	No. of Joints	Age, yr		FU, mo		Male Sex, %	Comparison Group	Diagnosis (n)
						Mean	Range	Mean	Range			
Andreozzi et al.^25^	2022	Italy	4	12	12	57∗	NR	12	-	75	NR	RA (6), PSA (6)
Annibaldi et al.^26^	2022	Italy	26	15	15	33	21-55	7.2	6-10	73	NR	Anterior cruciate ligament repair (15)
Colasanti et al.^27^	2022	US	24	31	31	42	17-69	15.5	±4.9	58	NR	Anterior ankle impingement (31)
DeClouette et al.^28^	2022	US	34	34	35	44	NR	NR	NR	65	NR	Rotator cuff tear, labral tear, loose body, meniscus, chondral (35)
Lopas and Mir^29^	2023	US	NR	4	5	57	47-74	NR	NR	50	NR	Anatomic reconstruction (5)
McMillan et al.^23^	2019	US	25	1,419	1,419	NR	14-78	24	NR	NR	NR	Intra-articular abnormalities such as joint swelling, pain, mechanical symptoms, and positive provocative physical examination test findings
Mercer et al.^30^	2022	US	12	10	10	42	24-66	13.3	11-17	40	NR	Posterior ankle impingement syndrome (10)
Moreland et al.^31^	1995	US	30	47	47	49	NR	NR	NR	34	NR	RA (30), OA (10), SA (2), OA/CCPD (1), UCTD (2), SpA (1), Reiter syndrome (1)
Schaver et al.^13^	2023	US	12	19	19	43	NR	1.5	-	47	Conventional arthroscopy	Partial meniscectomy (19)
Stornebrink et al.^24^	2021	NL	11	9	10	59	35-77	NR	NR	33	NR	SA (10)
Stornebrink et al.^32^	2022	NL	12	24	24	47	20-71	0.5	-	46	NR	OA (20), OCD (4)

NOTE. Deleted joints consisted of a hip joint (n = 1) (Lopas and Mir) and a wrist joint (n = 1) (Stornebrink et al.).

CCPD, calcium pyrophosphate dihydrate crystal deposition disease; FU, follow-up; NL, The Netherlands; NR, not reported; OA, osteoarthritis; OCD, osteochondritis dissecans; PSA, psoriatic arthritis; RA, rheumatoid arthritis; SA, septic arthritis; SpA, septic polyarthritis; UCTD, undifferentiated connective tissue disease; US, United States.

∗ Median. ± indicates the range of variability around a central value (e.g., the standard deviation).

The 11 studies reported on a total of 1,624 patients with 1,627 needle arthroscopic interventions. The mean age of patients was 46 years (range, 33-59 years), and 52% of patients were men (range, 33%-75%) (Table 2); none of the studies disaggregated the data by sex. The NanoScope (Arthrex, Naples, FL) was used in 73% of the studies (n = 8). Among the interventions, the most commonly used scope was the Mi-Eye 2 (Trice Medical, Malvern, PA), accounting for 87% of the procedures (1,419 from a single study). The remaining 3 scopes—IntraVu MIDASVu (Redwood City, CA), Medical Dynamics Fiberoptic (Nieuwegein, The Netherlands), and Trice Medical Mi-Eye—were each used in only 1 of the included studies (Table 3). Seventy-two percent of the interventions were performed under local anesthesia (Appendix Table 3).

**Table 3:** Complications of Needle Arthroscopy

Authors	Patients, n (%)	Joint (n)	Type of Needle Arthroscopy (n)	Complications
Andreozzi et al.^25^	12 (0.7)	Knee (12)	NanoScope (12)	Vasovagal reaction: 8.33% (n = 1)
Annibaldi et al.^26^	15 (0.9)	Knee (15)	NanoScope (15)	None
Colasanti et al.^27^	31 (1.9)	Ankle (31)	NanoScope (31)	Calcaneofibular ligament pain: 3.23% (n = 1) Nerve pain: 6.45% (n = 2)
DeClouette et al.^28^	34 (2.1)	Shoulder (25) Knee (10)	MIDASVu (35)∗	None
Lopas and Mir^29^	4 (0.2)	Ankle (4) Knee (1)	Mi-Eye (5)	None
McMillan et al.^23^	1,419 (87.4)	Shoulder (300) Knee (1,119)	Mi-Eye 2 (1,419)	Vasovagal reaction: 1.9% (n = 27) Persistent pain for 24 h: 0.28% (n = 4)
Mercer et al.^30^	10 (0.6)	Ankle (10)	NanoScope (10)	None
Moreland et al.^31^	47 (2.9)	Knee (47)	Medical Dynamics Fiberoptic (47)	None
Schaver et al.^13^	19 (1.2)	Knee (19)	NanoScope (19)	None
Stornebrink et al.^24^	9 (0.6)	Knee (8) Ankle (1) Shoulder (1)	NanoScope (10)	None
Stornebrink et al.^32^	24 (1.5)	Ankle (24)	NanoScope (24)	None

∗ Two patients were treated with the NanoScope.

**Fig 2:**
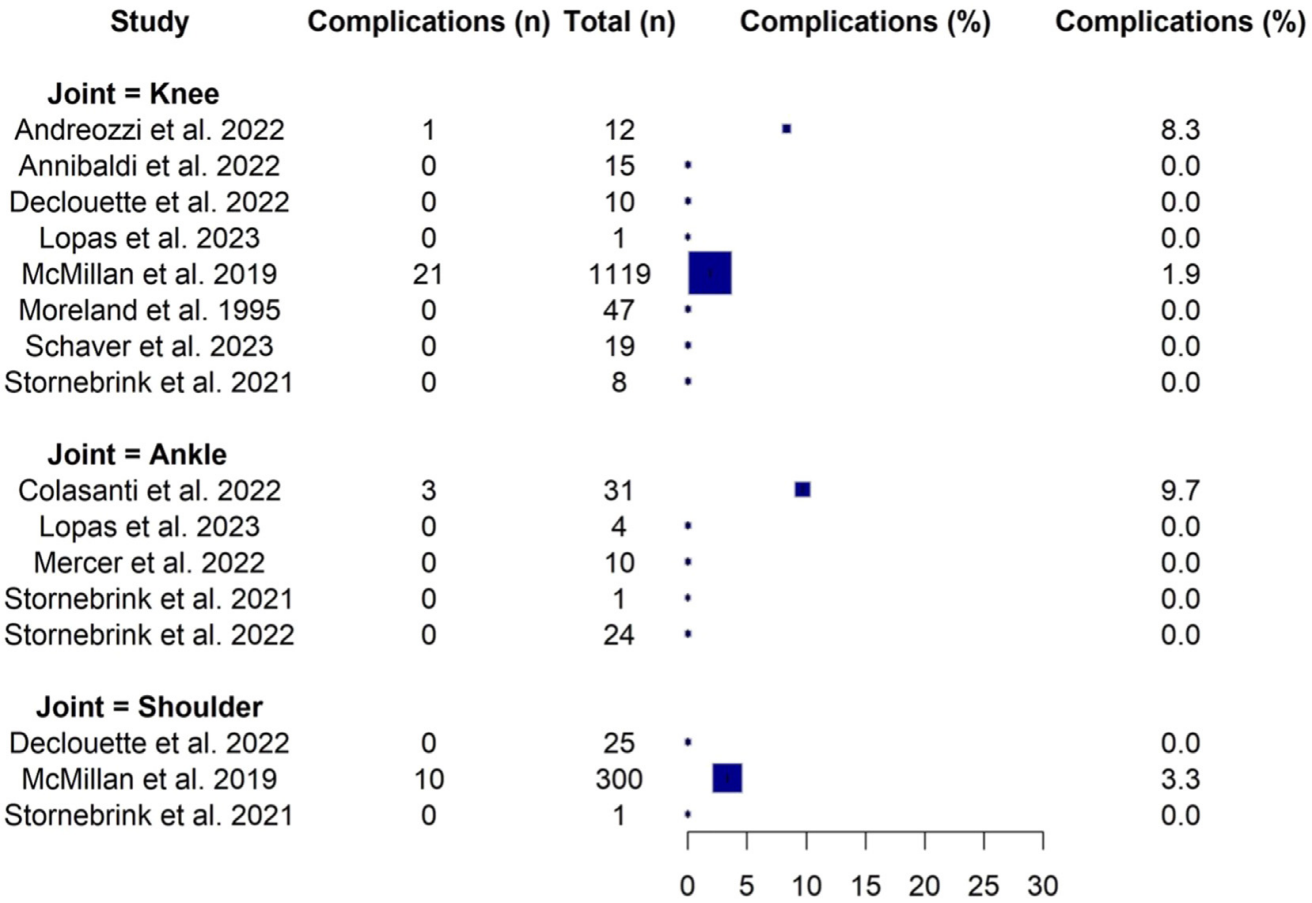
Forest plot of complications per joint.

Because of the lack of comparative studies, no pooling of data was conducted as recommended.^33^ The reported complication rates ranged from 0% to 9.68% (Fig 2, Appendix Table 4). Complications were observed after the use of needle arthroscopy in only 4 studies, whereas 7 studies reported no complications. The most frequently reported complication was vasovagal reaction (0%-8.33%). All complications were classified as grade I according to the Clavien-Dindo-Sink classification (Table 3).

## Discussion

In this study, we found that needle arthroscopy is a safe procedure with rates of complications ranging from 0% to 9.68%, all of which were minor (grade I). The most frequently reported complication was vasovagal reaction (0%-8.33%). This review underscores the safety of needle arthroscopy as an intervention. Given its smaller equipment and the ability to perform procedures in the office under local anesthesia, needle arthroscopy can be considered a useful alternative in the orthopaedic tool set.

Similar complication rates have been reported for conventional (4-mm) arthroscopy, with reported complication rates ranging from 1% to 11%.^15^^,^^16^^,^^34^ All complications of needle arthroscopy were classified as grade I, in contrast to conventional arthroscopy, where higher grades of complications occur.^15^^,^^16^ This may be because of its percutaneous and minimally invasive technique, which is aided by the use of a smaller scope than conventional arthroscopy. The inclusion criteria of this review specified that the arthroscopes used had a maximum diameter of 2.5 mm. This was based on the idea that modern-day needle arthroscopes have a diameter of around 2 to 2.5 mm and to exclude already existing small wrist arthroscopes (2.7 mm) used in the operation theater. This approach may cause less tissue injury, thus lowering the chance of problems. However, more rigorous comparative research is required to enable a more robust comparison.^35^

In our review, vasovagal reactions were the most frequent complication, which is mostly caused by needle phobia, which is a complication specific to needle arthroscopy and does not occur with conventional arthroscopy. Sokolowski et al.^36^ reported that 10% of the worldwide population has some form of needle phobia. Anxious patients are also more likely to experience a vasovagal reaction, although this decreases with age.^36^ Alsbrooks and Hoerauf^37^ reported that non–needle-related medical fears and family history of needle phobia were the most important factors associated with needle phobia. Additionally, Orenius et al.^38^ reported that 80% of patients with needle phobia indicated a significant fear of needles in a first-degree relative. Distractions and medical equipment–related education were perceived as the most helpful non–device-related strategies by participants.^37^ McMillan et al.^23^ suggested that substituting the term “needle arthroscope” with a more general name such as “small scope” or “camera” may be helpful, although no evidence was provided to substantiate this hypothesis. Another possible way to reduce anxiety is to turn the monitor away or reassure the patient. We believe that patient education could also play a crucial role in minimizing complications after needle arthroscopy. However, several studies have also highlighted the significance of the surgeon’s experience in performing these procedures. Given that these interventions are relatively new, it is reasonable to assume there is a learning curve associated with their execution. Through proper training and gaining experience beforehand, it is possible to decrease the occurrence of complications. Recently, multiple technique articles were published to learn how to use needle arthroscopy properly for different indications.^1^^,^^2^^,^^9^^,^^39^^,^^40^

Another identified complication was (nerve) pain after the intervention. Amadei et al.^41^ discovered that such pain is primarily caused by lesions resulting from direct nerve damage during portal creation or surgical maneuvers. Additionally, indirect (e.g., iatrogenic) damage can occur owing to traction or pressure mechanisms, particularly in cases of patient positioning errors. However, because of the smaller equipment and portals used in needle arthroscopy, far fewer complications related to these issues are observed than in conventional arthroscopy (the most frequently reported complications were neurologic injuries).^15^^,^^16^ This is particularly notable in the ankle, where the anterolateral portal poses a risk to the superficial peroneal nerve.^42^^,^^43^

Another important note is that none of the cases treated with needle arthroscopy resulted in joint infection (i.e., bacterial arthritis), a complication occurring in 1% to 5% of cases with conventional arthroscopy, despite the less sterile environment with in-office needle arthroscopy.^34^ It is interesting to note that a promising application of needle arthroscopy lies in the management of bacterial arthritis in native joints.^24^^,^^40^

The complication rates associated with the different brands of scopes examined in this review varied from 0% to 10%; however, most of these scopes were only assessed in a single study, with the smallest only encompassing 10 patients. This makes quantitative comparison between the brands not feasible.

Needle arthroscopy can be used in all appendicular joints, although our review focuses only on the knee, shoulder, and ankle. The complication rates per respective joint observed in our review are comparable to or better than those reported for conventional arthroscopy in the literature. Pajalic et al.^34^ reported a 1.1% absolute risk of complications, including pyogenic arthritis, venous thromboembolism, and other surgical issues, in a large cohort of 18,735 knee arthroscopy patients. None of these were observed in our studies. Complications after ankle arthroscopy were reported at rates ranging from 3% to 9%, with—as mentioned earlier—neurologic injury as the most common.^15^ Furthermore, rates of complications after shoulder arthroscopy ranged from 1.0% to 7.9%.^44^^,^^45^ Therefore, patients should be fully informed to make informed decisions between needle and conventional arthroscopy.

### Limitations

The findings of this study must be considered within the context of its limitations. First, one study contributed the majority of patients (n = 1,419), limiting the generalizability and consistency of conclusions. In contrast, the smaller studies are more susceptible to skewed results—one complication could equate to an 8.33% complication rate—and their lower statistical power further undermines the strength of the conclusions. Second, the quality of the included studies was heterogeneous, which may impact the reliability of the findings. Four studies were at low risk of bias, 5 were at moderate risk of bias, and 2 were at high risk of bias, highlighting significant concerns regarding study design, execution, and reporting that could compromise the robustness of their conclusions. This variability underscores the need for greater methodologic rigor in future research. Third, 9 of the 11 included studies used a retrospective methodology, which limits the strength of the evidence by introducing potential biases such as selection bias, recall bias, and the inability to adjust for confounding variables. Although retrospective studies might provide useful initial data, they are not well adapted to establishing connections between variables or validating safety and efficacy. The current literature is dominated by retrospective studies, indicating a lack of high-quality evidence. To confirm the safety and efficacy of emerging minimally invasive needle arthroscopy procedures, well-designed prospective research and randomized controlled trials are required.

This review shows that the safety of needle arthroscopy as an intervention is factory in the literature to date, with seemingly less severe complications than the literature on conventional arthroscopy. Given its smaller equipment and the ability to perform procedures in the office under local anesthesia, needle arthroscopy appears to be a useful alternative in the orthopaedic tool set for particular indications. Continued training and practice enable orthopaedic surgeons to refine their skills in these procedures, potentially enhancing their utility. Proper instructions of patients are warranted to reduce anxiety and related complications. Various technical notes have been published to aid familiarity with needle arthroscopy.

## Conclusions

In this study, we found that the reported complication rate after needle arthroscopy ranged from 0% to 9.68%. All reported complications were classified as grade I according to the Clavien-Dindo-Sink classification.

## Disclosures

The authors declare the following financial interests/personal relationships which may be considered as potential competing interests: Support was provided by the Dutch Arthritis Society, Dutch Research Council (KIC [Knowledge and Innovation Covenant]: early detection of osteoarthritis), and Netherlands Organisation for Health Research and Development (Health Care Efficiency Research). A.B.W. receives funding grants from Netherlands Organisation for Health Research and Development. G.M.M.J.K. receives funding grants from Netherlands Organisation for Health Research and Development and Arthrex GmbH and reports a consulting or advisory relationship with Arthrex GmbH. K.S.E. receives funding grants from Netherlands Organisation for Health Research and Development and Dutch Research Council. The other author (T.v.d.S.) declares that they have no known competing financial interests or personal relationships that could have appeared to influence the work reported in this paper.
