# Supplementary material for: Only Minor Complications Are Reported After Needle Arthroscopy: A Systematic Review
Source: Arthrosc Sports Med Rehabil. 2025 Apr 28;7(4):101158. doi: 10.1016/j.asmr.2025.101158 (PMC12447137; doi:10.1016/j.asmr.2025.101158)
Supplement: Supplementary Tables [file mmc1.pdf]

Appendix Table 1. Full Search Syntax Per Library

No. or ID	Search	Hits
PubMed		
1	("Arthroscopy"[Mesh] AND "Needles"[Mesh]) OR (needle arthroscop*[tiab] OR in-office arthroscop*[tiab] OR Trice Medical[tiab] OR Needle-based diagnostic arthroscop*[tiab] OR VSI system[tiab] OR VisionScop*[tiab] OR Vision scop*[tiab] OR Office based arthroscop*[tiab] OR Bed side needle arthroscop*[tiab] OR Bedside needle arthroscop*[tiab] OR disposable needle scope[tiab] OR diagnostic scope system*[tiab] OR IONA[tiab] OR Nano arthroscop*[tiab] OR Nanoarthroscop*[tiab] OR Small-bore needle arthroscop*[tiab] OR nanoscope[tiab])	403
Embase (Ovid)		
1	arthroscopy/ and needle/	155
2	(needle arthroscop* or in-office arthroscop* or Trice Medical or Needle-based diagnostic arthroscop* or VSI system or VisionScop* or Vision scop* or Office based arthroscop* or Bed side needle arthroscop* or Bedside needle arthroscop* or disposable needle scope or diagnostic scope system* or IONA or Nano arthroscop* or Nanoarthroscop* or Small-bore needle arthroscop* or nanoscope).ti,ab,kf.	444
3	3 (needle* adj6 arthroscop*).ti,ab,kf.	320
4	1 or 2 or 3	681
Cochrane Central Register of Controlled Trials		

#1	MeSH descriptor: [Arthroscopy] explode all trees	1,977
#2	MeSH descriptor: [Needles] explode all trees	1,407
#3	#1 and #2	5
#4	(needle* near/6 arthroscop*):ti,ab,kw	22
#5	(office* near/6 arthroscop*):ti,ab,kw	9
#6	((bedside* or bed side*) near/6 arthroscop*):ti,ab,kw	106
#7	(disposable needle scope):ti,ab,kw	3
#8	(diagnostic scope system*):ti,ab,kw	128
#9	(IONA or nano arthroscop* or nanoarthroscop* or nanoscope):ti,ab,kw	14
#10	#3 or #4 or #5 or #6 or #7 or #8 or #9 in Trials	167

Appendix Table 2. Full Evaluation of ROBINS-I Tool

**Authors**	**D1**	**D2**	**D3**	**D4**	**D5**	**D6**	**D7**	**Overall**
Andreozzi et al.[25] (2022)	+	+	+	+	+	+	+	Low
Annibaldi et al.[26] (2022)	–	±	+	+	+	+	+	Moderate
Colasanti et al.[27] (2022)	–	±	+	+	+	+	+	Moderate
DeClouette et al.[28] (2022)	–	±	+	+	+	±	+	Moderate
Lopas and Mir[29] (2023)	–	–	+	+	–	+	–	High
McMillan et al.[23] (2019)	+	+	+	+	+	+	+	Low
Mercer et al.[30] (2022)	–	±	+	+	+	+	+	Moderate
Moreland et al.[31] (1995)	+	±	+	±	+	+	+	Moderate
Schaver et al.[13] (2023)	–	±	+	+	+	±	+	High
Stornebrink et al.[24] (2021)	+	+	+	+	+	+	+	Low
Stornebrink et al.[32] (2022)	+	+	+	+	+	+	+	Low

NOTE. A plus sign indicates low risk; plus-minus sign, moderate risk; and minus sign, high risk. D1, bias due to confounding; D2, bias due to selection of participants; D3, bias in classification of interventions; D4, bias due to deviations from intended intervention; D5, bias due to missing data; D6, bias in measurement of outcomes; D7, bias in selection of reported results; ROBINS-I, Risk of Bias in Non-randomized Studies of Interventions.

Appendix Table 3. Patient Satisfaction and Procedural Insights of Needle Arthroscopy

Authors	Type of Needle Arthroscopy (n)	Intervention	Anesthesia (n)
Andreozzi et al.[25]	NanoScope (12)	Biopsy	Local
Annibaldi et al.[26]	NanoScope (15)	Inspection of anterior cruciate ligament	Local
Colasanti et al.[27]	NanoScope (31)	Debridement	Local
DeClouette et al. [28]	NanoScope (2), MIDASVu arthroscope (32)	Inspection	Sedation
Lopas and Mir[29]	Mi-Eye (5)	Fracture reduction	General
McMillan et al.[23]	Mi-Eye 2 (1,419)	Diagnostic	Local
Mercer et al.[30]	NanoScope (10)	Debridement	Local
Moreland et al.[31]	Medical Dynamics Fiberoptic (47)	Inspection/debridement	Local
Schaver et al.[13]	NanoScope (19)	Meniscectomy	General
Stornebrink et al.[24]	NanoScope (10)	Joint lavage	Local (8), general (2)
Stornebrink et al.[32]	NanoScope (24)	Injection	Local

Appendix Table 4. Complications per Joint

		Complications	
**Joint**	**Total, n (%)**	**n (%)**	**Range, %**
Knee	1,231 (75.7)	22 (1.79)	1.9-8.33
Shoulder	326 (20.0)	10 (3.07)	0.0-3.3
Ankle	70 (4.3)	3 (4.29)	0.0-9.68
